# Silver Nanocoatings for Reducing the Exogenous Microbial Colonization of Wound Dressings

**DOI:** 10.3390/ma9050345

**Published:** 2016-05-06

**Authors:** Marius Radulescu, Ecaterina Andronescu, Georgiana Dolete, Roxana Cristina Popescu, Oana Fufă, Mariana Carmen Chifiriuc, Laurenţiu Mogoantă, Tudor-Adrian Bălşeanu, George Dan Mogoşanu, Alexandru Mihai Grumezescu, Alina Maria Holban

**Affiliations:** 1 Department of Inorganic Chemistry, Faculty of Applied Chemistry and Materials Science, University Politehnica of Bucharest, 1–7 Polizu Street, Bucharest 011061, Romania; radulescu_marius@yahoo.com; 2 Department of Science and Engineering of Oxide Materials and Nanomaterials, Faculty of Applied Chemistry and Materials Science, University Politehnica of Bucharest, 1–7 Polizu Street, Bucharest 011061, Romania; ecaterina.andronescu@upb.ro (E.A.); dolete.georgiana@gmail.com (G.D.); roxpopescu@yahoo.co.uk (R.C.P.); oana.fufa@gmail.com (O.F.); alina_m_h@yahoo.com (A.M.H.); 3 Department of Life and Environmental Physics, “Horia Hulubei” National Institute of Physics and Nuclear Engineering, Magurele 077125, Romania; 4 Lasers Department, National Institute for Laser, Plasma and Radiation Physics, Magurele 077125, Romania; 5 Microbiology Immunology Department, Faculty of Biology, Research Institute of the University of Bucharest (ICUB), University of Bucharest, 1–3 Portocalelor Lane, Sector 5, Bucharest 77206, Romania; carmen_balotescu@yahoo.com; 6 Research Center for Microscopic Morphology and Immunology, University of Medicine and Pharmacy of Craiova 2 Petru Rareş Street, Craiova 200349, Romania; editor@rjme.ro; 7 Research Center for Clinical and Experimental Medicine, University of Medicine and Pharmacy of Craiova 2 Petru Rareş Street, Craiova 200349, Romania; adibalseanu@yahoo.com; 8 Department of Pharmacognosy & Phytotherapy, Faculty of Pharmacy, University of Medicine and Pharmacy of Craiova 2 Petru Rareş Street, Craiova 200349, Romania; mogosanu2006@yahoo.com

**Keywords:** silver nanoparticles, *Staphylococcus aureus*, *Pseudomonas aeruginosa*, antibacterial activity, coated wound dressings, biofilm inhibition

## Abstract

The aim of this work was to obtain an antimicrobial coating (NanoAg) for polyester-nylon wound dressings (WDs) for reducing the risk of exogenous wound related infections. The as-prepared NanoAg-WDs were characterized by XRD (X-ray Diffraction), SEM (Scanning Electron Microscopy), TEM (Transmission Electron Microscopy), SAED (Selected Area Electron Diffraction) and IRM (InfraRed Microscopy). Biological characterization consisted of *in vitro* evaluation of the interaction with fibroblast cell cultures and *in vivo* biodistribution studies of AgNPs on mice models. Then, specimens of commercial WDs were immersed in a glucose and NaOH solution of silver nanoparticles, followed by the subsequent dropwise addition of AgNO_3_ solution. The antimicrobial efficiency of the NanoAg-WDs was assessed by *in vitro* qualitative and quantitative analyses on *Staphylococcus aureus* and *Pseudomonas aeruginosa* strains. The *in vitro* and *in vivo* studies demonstrated that the tested nanoparticles utilized to coat WDs have a good biocompatibility, allowing the normal development of cultured human cells and revealing a normal biodistribution within a mouse model, without toxic effects. The modified and viable cells count analyses proved that the modified WDs exhibit an improved inhibitory activity of microbial colonization, attachment and biofilm growth. The reported data recommend this type of coatings to obtain modified WDs with antibacterial properties, able to prevent the exogenous microbial contamination of the wound tissue, colonization and further biofilm development.

## 1. Introduction

The correct care and treatment of a wound represents the key aspect to facilitate healing and to prevent infection, which could delay the process or even cause serious complications [1,2].

Burn patients, individuals in critical care units that undergo major surgery and immunocompromised patients are the most prone to acquiring acute skin and wound infections with opportunistic pathogens. In addition, chronic wound infections affect more than a half of patients with chronic skin injuries related to severe diseases, such as diabetes, and such infections include one or more microbial species, associated in biofilm consortia. Biofilms developed on the wounds and skin injuries of such patients are very difficult to eradicate and the current treatment involves a multi-oriented approach including the mechanical debridement, the use of wound dressings to limit the spread of infection and to facilitate healing, application of local disinfectants and specific antimicrobial therapy [3]. However, despite this multi-oriented approach, more than 70% of biofilm-associated wound infections become chronic conditions, presenting a high risk of sepsis, debilitating diseases and failure in the treatment of the primary diseases in diabetic patients [4].

Wound contaminants can originate from the environment, the surrounding skin microbiota (including the opportunistic pathogens *Staphylococcus (S.) aureus* and *Pseudomonas (P.) aeruginosa*) and the endogenous microbiota of mucous membranes [5,6,7,8,9].

The risk of infection is influenced by the type of wound dressing and is usually increased by inappropriate wound care, such as time elapsed between bandages change. The extent of nonviable exogenous contamination influencing the type of microbial load and the synergic level of virulence expressed by the different types of microorganisms involved that usually colonize the wounds in polymicrobial associations influence the progression of a wound to an infected state [10,11,12].

Therefore, in order to minimize the risk of wound infection and exclude exogenous microorganisms that could delay healing in non-infected wounds, controlling the microbial load in wounds is a vital factor that could be achieved by systemic or topical antibiotic therapy, antiseptics or other non-antimicrobial treatment methods [10].

Given their intrinsic biocide activity silver-based materials have been used for years as antimicrobials for the treatment of burns, traumas and diabetic ulcers [13,14,15,16]. Various silver- based products have become efficient alternatives in burn care, when compared to the commercial available antibiotics. Silver nanoparticles have been reported to have an improved efficiency against a broad range of microorganisms (including bacteria and fungi) and viruses [17,18,19]. The possible mechanisms of their antimicrobial activity are the alteration of structure of the cell membrane selective permeability, interaction with sulfur and phosphorus groups of many vital enzymes, generation of increase levels of reactive oxygen species [20,21,22].

The present approach aims to reduce the microbial load of a wound resulted from the attachment and growth of exogenous bacteria by using modified wound dressings resistant to microbial colonization, obtained by the immersion of commercial polyester-nylon dressings, regularly used in wound care, in silver nanoparticles coating solution. This is a non-toxic, simple, inexpensive and fast method to obtain antimicrobial WDs able to reduce microbial colonization, and inhibit growth and biofilm formation within the wound.

## 2. Results

### 2.1. Characterization of NanoAg-WDs

**Figure 1:**
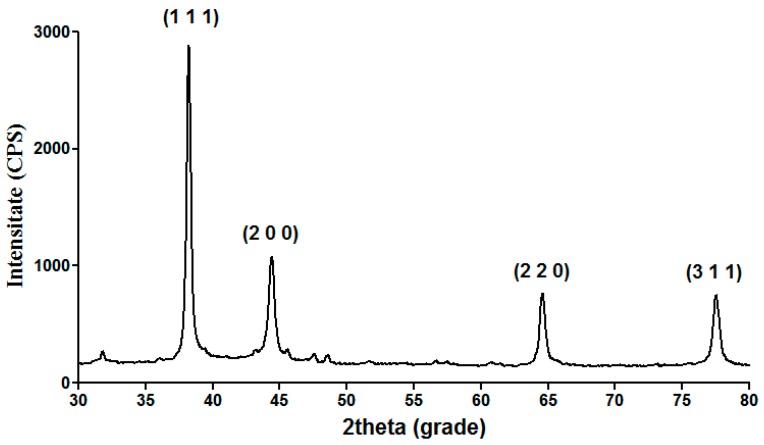
XRD pattern of NanoAg-WDs.

XRD (X-ray diffraction) analysis was performed in order to confirm the crystalline nature and the purity of the silver nanoparticles experimentally obtained by employing the chemical reduction method. The diffraction pattern shown in Figure 1 indicates the presence of specific Bragg reflections related to a face-centered-cubic structure of silver, according to the Joint Committee on Powder Diffraction Standards file No. 04-0783 and ASTM (American Society for Testing and materials, West Conshohocken, PA, USA) available charts. No other crystalline phases were identified, confirming thus the high purity of the obtained silver-based structures. In addition, considering the sharp aspect and the reduced width of the obtained diffraction peaks, we can correlate the reduced dimensions to the structural crystallites and to the following silver particles.

**Figure 2:**
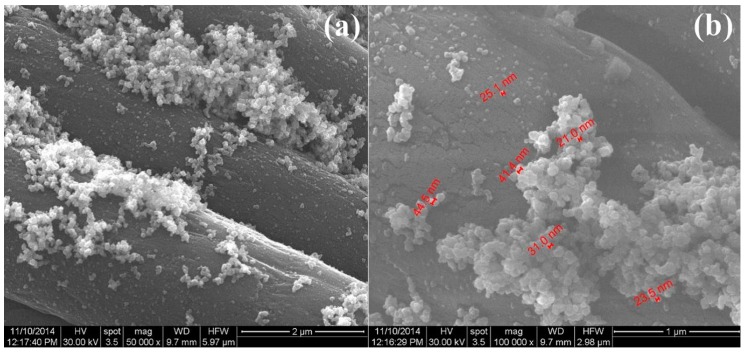
SEM images of NanoAg-WDs at various magnifications: (**a**) 50,000×; and (**b**) 100,000×.

The morphology of the silver-based dressings was evaluated using the scanning electron microscopy (SEM) analysis. The most representative SEM images are presented in Figure 2. It can be noticed that the silver nanoparticles have an irregular distribution onto the dressing fibers. The metallic particles also possess an increased tendency to form agglomerates, especially at fiber junctions. This behavior may be assigned to the specific affinity between the higher surface energy of the AgNPs and the increased strains established among contiguous dressing fibers. At higher magnification (Figure 2b), the spherical morphology of the synthesized silver nanoparticles can be observed. By considering this magnification range and the accompanied specific software of the microscope, we were able to evaluate the dimensions of the AgNPs, which possess sizes ranging from 20 nm to 50 nm.

**Figure 3:**
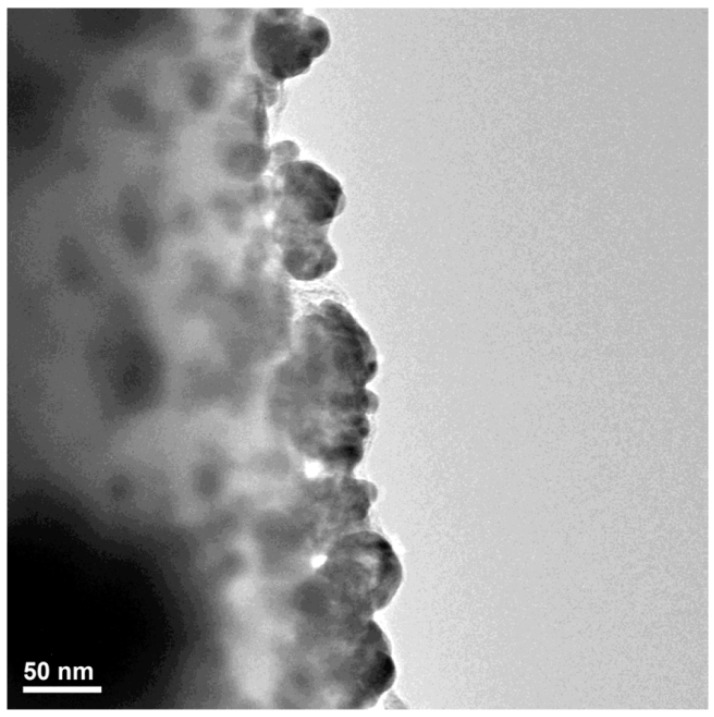
TEM bright field image of NanoAg-WDs.

**Figure 4:**
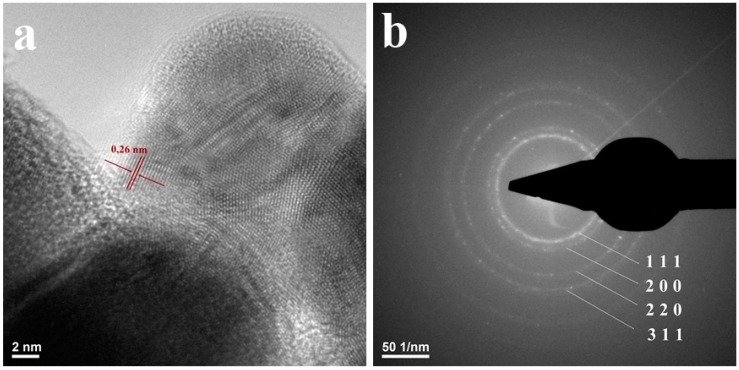
(**a**) HR-TEM image; and (**b**) SAED pattern of NanoAg-WDs.

Transmission electron microscopy (TEM) images confirm our previous studies regarding the nanosized dimensions of the obtained silver particles (lower than 50 nm) and their spherical shape (Figure 3).The increased tendency of nanosilver to form agglomerate structures was also confirmed. By using High Resolution TEM (HR-TEM) imaging, the high crystallinity of the Ag nanoparticles was confirmed and an interplanar distance of 2.4 Å was measured (Figure 4a).The obtained SAED spectrum presented in Figure 4b confirmed the XRD data regarding the face-centered cubic crystalline structure of the synthesized AgNPs, the as-obtained concentric rings corresponding to the Bragg (1 1 1), (2 0 0), (2 2 0) and (3 1 1) diffraction planes.

**Figure 5:**
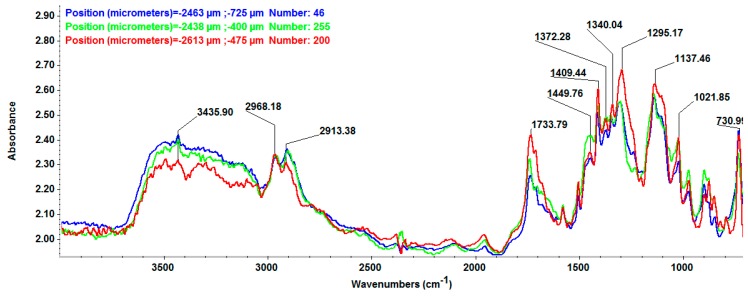
InfraRed spectra of NanoAgWDs.

**Figure 6:**
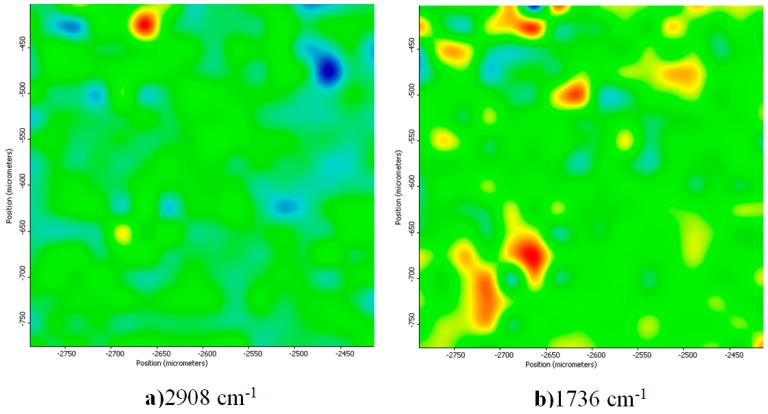
IR maps of NanoAgWDs: Intensity distribution of: (**a**) 2908 cm^−1^ (CH_3_); and (**b**) 1736 cm^−1^ (C=O) wavenumbers.

The compositional integrity of the functional groups of the dressings after the synthesis process was observed by infrared microscopy (IRM) means. As seen in Figure 5, the IR spectra obtained for the nanosilver-based wound dressings present typical absorption peaks assigned to both polyester-nylon fibers and d-glucose reducing agent. The specific positions of the absorption peaks assigned to the composite fibers have not been altered during the synthesis process, thus suggesting that surface physical and weak chemical interactions occurred between the commercial fibers and the synthesized AgNPs. The absorption data with respect to the –CH_3_ stretching vibrations and the C=O rotation vibrations were further considered when building the IR maps that are presented in Figure 6. As the infrared maps show, we obtained a structural and compositional homogenous material. By correlating this information with the classical IR analysis, it can be concluded that, after the reduction of silver ions onto the surface of the commercial dressings, no damaged functional groups or chemical structural changes appeared.

### 2.2. Biological Evaluation

#### 2.2.1. Biocompatibility of Silver Nanoparticles Coating Solution

Presently, there is no available WD that meets all specific requirements for the development of efficient post-operative wound dressings, *i.e.*, high moisture vapor permeability maintenance, barrier-like activity for water and bacteria, trauma-free dressing removal and physiological unnoticeable adhesion [23]. Nonetheless, the designed wound dressing system should also be non-toxic, non-allergic, non-adherent and sterile, like any other medical device that interacts with the patient.

Silver-containing dressings may be successfully used for acute wounds treatment, such as surgical or traumatic wounds (including burns) and for chronic wounds treatment, including localized open or closed injuries, preventing thus microbial spread and subsequent systemic infection [24]. It is well-known and experimentally proved that metallic and metal oxide nanoparticles of smaller sizes induce an increased toxic potential against cells, because of their tendency to release metallic ions within the physiological environment [25,26,27]. Therefore, the silver nanoparticles coating solution was tested by *in vivo* and *in vitro* assays for its biocompatibility before being used for obtaining the modified WD.

#### 2.2.2. *In Vitro* Study of Biocompatibility

The quantitative and qualitative evaluations of cell proliferation for AgNPs were done in order to follow the cells behavior depending on the concentration of the nanoparticles suspension (1.5 and 10 μg/mL).

**Figure 7:**
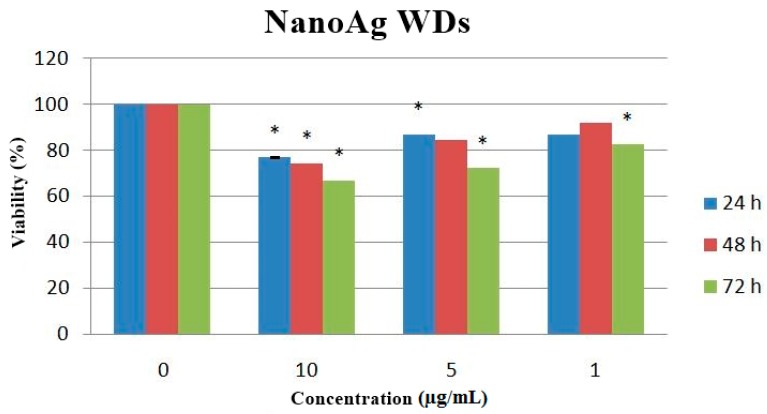
Cell viability depending on AgNPs concentration. * *p* < 0.05.

As can be observed in Figure 7, there is a descending trend of cell viability in time for the same concentration (except the 1 μg/mL concentration), as revealed by the MTT assay. According to ASTM standards for biocompatibility evaluation, for 1 and 5 µg/mL Ag nanoparticles concentrations, the nanoparticles utilized to obtain the improved wound dressings are non-cytotoxic.

**Figure 8:**
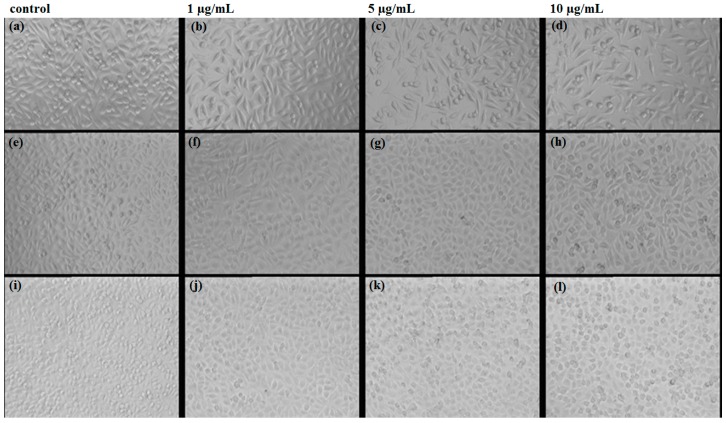
Images of optic microscopy at 24 (**a**–**d**); 48 (**e**–**h**) and 72 h (**i**–**l**) of the L929 cells grown in the presence of the obtained AgNPs.

The qualitative evaluation was determined on the L929 cell line after 24/48/72 h of exposure using phase contrast microscopy. Cell morphology was not changed in any of the observation intervals. Compared with the control, a cell number decrease is observed, more severely for the 10 µg/mL (Figure 8).

**Figure 9:**
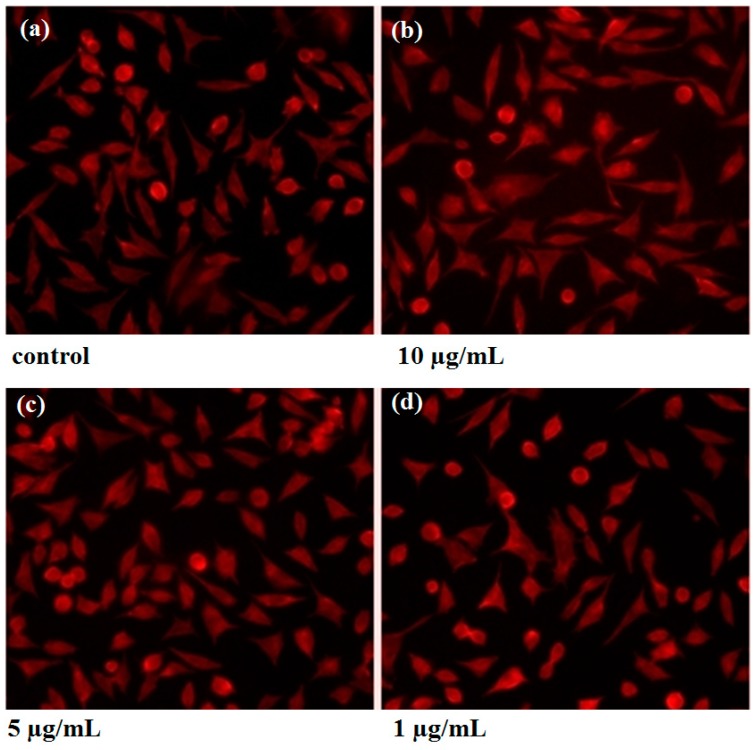
Images of fluorescence microscopy at 24 h of the L929 cultured cells grown in the presence of AgNPs: (**a**) control; (**b**) 10 μg/mL; (**c**) 5 μg/mL; (**d**) 1 μg/mL.

By using fluorescence imaging, we emphasized that the cytoskeleton integrity is not affected after the cells interaction with the AgNPs. The cell detachment is not visibly enhanced by increasing the concentration of silver nanoparticles, neither morphological changes, nor actin displacement can be observed (Figure 9).

#### 2.2.3. *In Vivo* Biodistribution

*In vivo* assay revealed a distinct biodistribution of the AgNPs depending on the type of tissue and time after the treatment.

**Figure 10:**
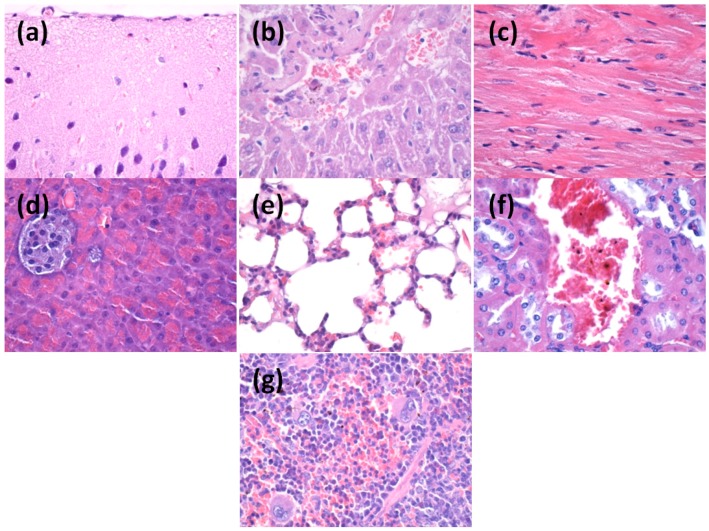
Transversal section through: (**a**) brain; (**b**) liver; (**c**) myocardium; (**d**) pancreas; (**e**) lungs; (**f**) kidneys; and (**g**) spleen from mice injected with AgNPs; samples collected at two days after the treatment; Hematoxylin-Eosin coloring (400× magnification, Nikon Instruments, Bucharest, Romania).

After two days post-treatment, microscopy evaluation of the organs sections revealed that nanoparticles are absent in the brain, myocardium and pancreas tissue. However, several aggregates containing silver nanoparticles were revealed in the liver, kidney and spleen tissues (Figure 10).

In the liver, a low amount of nanoparticles was observed, usually along blood vessels irrigating the hepatic tissue but also in the Kuppfer cells around the sinusoid capillary. The density of nanoparticles was variable in the Kuppfer cells, being proportionally with the diameter of capillary in the hepatic parenchim (Figure 10).

Similarly, after two days of treatment, silver nanoparticles were observed in the pulmonary tissue, especially in the perivascular macrophages and within intraalveolar septums. The density of nanoparticles was different depending on the type and distribution of the cells. The highest density of nanoparticles was observed in perivascular macrophages, while the lowest amount was seen in the intraalveolar septums. Nanoparticles were also detected within intravascular monocites. It seems that monocites are able to engulf silver nanoparticles and this could explain the presence of nanoparticles in the vascular lumen. In the vascular lumen we also observed the presence of nano aggregates outside the blood cells but also in the trombocites. In addition, we speculate that within the blood flow there may exist some substances that may act as transporters for nanoparticles and are able to agglutinate nanoparticles in the working conditions.

In the kidney, after two days post-treatment, we observed low amounts of nanoparticles, mainly around blood vessels. In other areas of renal tissue no nanoparticles were detected.

Similarly, in the spleen, we could detect nanoparticles only within the red pulp, while in the area no nanoparticles were detected. However, microscopy examination revealed a hypertrophy of white pulp, possibly because silver nanoparticles were able to stimulate the macrophages with multi-lobe nucleus. In the red pulp, most nanoparticles were observed after engulfment in the macrophages situated in Billroth lanes and also sinusod capillary. Nanostructures are visible as dark granular structures forming different sized clusters, with the average size of 3 μm.

**Figure 11:**
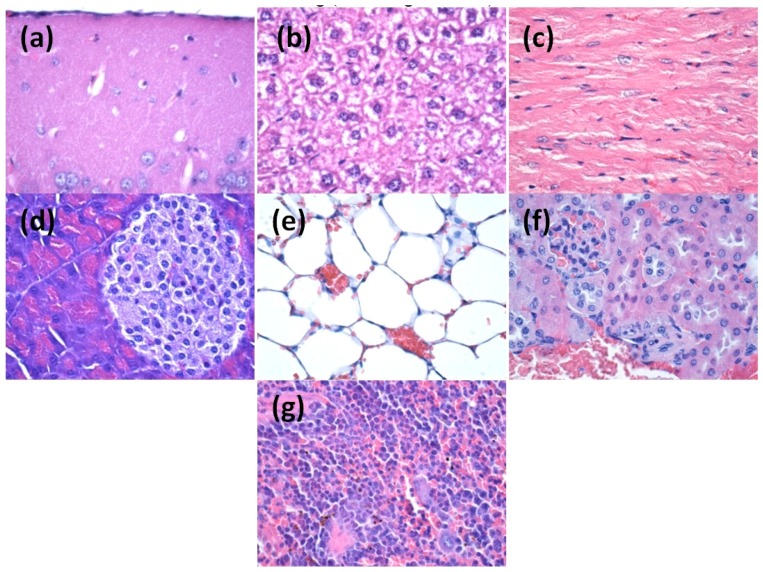
Transversal section through: (**a**) brain; (**b**) liver; (**c**) myocardium; (**d**) pancreas; (**e**) lungs; (**f**) kidneys; and (**g**) spleen from mice injected with AgNPs; samples collected at 10 days after the treatment; Hematoxylin-Eosin coloring (400× magnification).

After 10 days of treatment, the distribution of the nanoparticles within the analyzed organs was slightly different (Figure 11). Most nanoparticles were eliminated from the mouse body and we could not detect any nanoparticles within brain, liver, miocard, pancreas, lungs and kidneys. However, nanoparticles were still present in the spleen, where different sized clusters were observed.

Microscopy analysis of spleen sections revealed that after 10 days post-treatment nanoparticles are still clustered within the red pulp. Moreover, the amount of nanoparticles in the red pulp was higher as compared with tissue samples obtained after two days of treatment. Similar to the results obtained after two days of treatment, nanoparticles were absent within the white pulp, but this area presented extensive hypertrophy and numerous macrophages.

#### 2.2.4. Antibacterial and Anti-Biofilm Activity of Modified WDs

Biofilms are mono specific or poly specific microbial communities composed of microbial cells attached to an inert or viable substrate, which is protected by a complex polysaccharide matrix and confer to individual cells new properties, different of their free floating counterparts. Biofilms are highly tolerant to high amounts of antibiotics and antimicrobial drugs, therefore many biofilm associated infections are considered unhealable [28]. Along with the intrinsic tolerance of biofilms to antibiotic therapy, resistant pathogenic bacteria represent an additional inquiry in antimicrobial therapy [29,30,31,32,33]. The alarming increase of multi-drug resistant bacterial strains and difficult to treat biofilm related infections in wounds require the development of new wound care approaches and therapeutic strategies. One of the current focusesin the field is to design smart dressings able to limit microbial contamination of the wound and to inhibit attachment, growth and biofilm formation on the site of the skin lesion. The approached study allowed us to evaluate both short-term antimicrobial activity and long-term efficiency of the prepared WDs against bacterial biofilm development [34].

**Figure 12:**
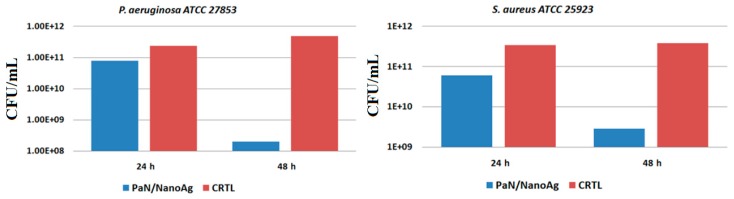
Graphic representation of the results obtained after CFU/mL count for *P. aeruginosa* and *S. aureus* biofilms developed in the presence of regular (control) and nano-coated WDs.

The experimentally obtained data with respect to antibacterial activity of nanosilver-based wound dressing are presented in Figure 12. For the control samples treated with *P. aeruginosa* bacterial strain, we noticed an increased tendency of the microorganisms to proliferate and produce biofilms by increasing the number of colony forming units (CFU) proportionally with the elapsed time, after 24 and 48 h of experimental treatment. When considering the silver-modified wound dressings, a slight decrease of the CFU/mL is reported after 24 h of incubation, whereas a significant reduction of biofilms formation and maturation with more than two orders of magnitude is reported after 48 h of experimental treatment. We concluded that the obtained results are clinically relevant, if we consider the increased acknowledged affinity of *Ps. aeruginosa* for medical device contamination, colonization and biofilm formation, our results being in accordance with previously published studies [35].

In *S. aureus*, it is worthy to mention that the CFU/mL values are higher than those reported for the previously discussed Gram-negative bacterial strain, thus suggesting a slight resistance of this Gram-positive bacterium when treated with AgNPs. In the case of biofilms produced by *S. aureus* after 24 h of biofilm development, the CFU/mL values (obtained after counting the viable cells released from mature biofilms grown in the presence of WDs coated with silver nanoparticles) reported a 10 times decrease in value, whereas a 20 times decrease of the CFU/mL count was observed after 48 h of bacterial incubation.

## 3. Materials and Methods

### 3.1. Materials

Silver nitrate, d-glucose and sodium hydroxide were purchased from Sigma-Aldrich. The chemicals were of analytical reagent grade and they were used without further purification. The required aqueous solutions were prepared by using distilled deionized water (DDW).

The experimentally modified wound dressings consisted of commercial polyester-nylon dressings, provided by a local supplier.

### 3.2. Synthesis of Nanosilver-Based Dressings

The experimental fabrication of silver-based dressings was carried out using a simple chemical process. Thus, the purchased commercial polyester-nylon dressings were cut into 1 cm^2^ sections. The as-obtained sections were further immersed into a solution consisting of 1 g of d-glucose, 4 g of NaOH and 400 mL of double distilled water (DDW) mixture.

The metallic precursor solution was obtained by dissolving 0.1 g of AgNO_3_ in 100 mL of DDW. The silver-containing solution was further drop wise added to the previously prepared mixture, under vigorous magnetic stirring.

The as-modified silver-based dressings were subsequently washed with DDW (in order to remove the exceeding reagents) and dried at room temperature.

### 3.3. Characterization of the Nanosilver-Based Dressings

#### 3.3.1. X-ray Diffraction

In order to experimentally examine the purity and crystallinity of the silver nanoparticles synthesized within the concerned wound dressings, we performed the diffraction analytical technique using a Schimadzu XRD 6000 diffractometer (Shimadzu, Kyoto, Japan) with Cu_Kα_ radiation (λ = 1.056 Å). The sample scanning was made by using a 2θ scattering angle between 10° and 80°.

#### 3.3.2. Scanning Electron Microscopy

Relevant data with respect to the morphological and compositional features of the experimental samples were acquired using Scanning Electron Microscopy (SEM means). Thus, for the modified polyester-nylon samples, the SEM analysis was performed using a FEI scanning electron microscope (FEI, Hillsboro, OR, USA), using the secondary electron beam with 30 keV energy.

#### 3.3.3. Transmission Electron Microscopy

The intimate microstructure of the obtained materials was investigated using Transmission electron microscopy (TEM) analysis. The concerned data were acquired using a Tecnai™ G2 F30 S-TWIN high resolution transmission electron microscope equipped with SAED instrument, purchased from FEI Company (FEI, Hillsboro, OR, USA)

#### 3.3.4. Infrared Microscopy

The IR mappings were recorded in the reflection mode by considering the 4000–600 cm^−1^ measurement range at 4 cm^−1^ resolution of a Nicolet iN10 MX FT-IR Microscope, equipped with MCT liquid nitrogen cooled detector. For each spectrum, 32 distinctive scans were recorded, co-added and converted to absorbance using the OmincPicta Software (Thermo Scientific, Walthman, MA, USA). An approximate number of 250 distinctive spectra were analyzed for each sample. Two absorptions peaks known as being characteristics for AgNPs were selected as specific spectral markers.

### 3.4. Biological Evaluation of Nanosilver-Based Dressings

#### 3.4.1. *In Vitro* Biocompatibility

The *in vitro* biocompatibility was evaluated on L929 fibroblast cell cultures, by employing the tetrazolium-based viability assay (MTT) to quantitatively assess the cell proliferation, whereas the morphology was observed using the optical microscopy technique for living cells. Fluorescence staining using Phalloidin, which specifically binds to filamentous actin was performed to verify the cytoskeleton integrity of the treated cells.

L929 cell line is highly recommended for cytotoxicity studies, due to its high sensibility. The cells were cultured in MEM Earle’s (MEM) (Biochrom, Merck Milipore, Berlin, Germany), supplemented with 10% fetal bovine serum (Biochrom, Merck Milipore), 1% l-glutamine (Biochrom, Merck Milipore) and 1% antibiotics (penicillin and streptomycin) (Biochrom, Merck Milipore).

For the MTT assay, 5000 cells/well were seeded in 96-well plates and cultured for 24 h in standard conditions (37 ± 2 °C, 5% ± 1% CO_2_, more than 90% humidity). Meanwhile, solutions of nanoparticles in different concentrations (10, 5, and 1 µg/mL) were prepared by ultrasound dispersion in complete culture medium and added in each of the sample wells, while in control wells (untreated cells) complete culture medium was added; blank samples were also prepared (wells with no cells) in order to eliminate possible interferences. The viability measurements were done at 24, 48, and 72 h after the treatment: the nanoparticles were gently removed from the wells and 10 µL of MTT solution was added over 90 µL complete culture medium (supplemented with 5% Fetal Bovine Serum) in each well. The plates were incubated for 2 h in standard conditions. After this time, 100 µL of acid isopropyl alcohol solubilization solution was added in each well and plates were subjected tovigorous shaking for several minutes. The absorbance was read at 570 nm using Mitras LB 940 (Berthold Technologies, Calmbacher, Germany).

For the morphology evaluation using optical microscopy, the images were recorded using an Olympus CKX31CF microscope, from the samples prepared for the viability assay, prior to the addition of the MTT solution.

For the qualitative evaluation of the cytotoxic effects, we evaluated the morphology and cytoskeleton integrity of treated cells using Texas Red^®^-X Phalloidin (ThermoFisher Scientific) fluorescence staining. For this purpose, 5000 cells were seeded on each 10 mm diameter glass slides placed in 24-well plates and cultured for 24 h in standard conditions. The treatment was done similarly to the cytotoxicity assay. At 24 h after the treatment, the nanoparticles were removed from the wells and the cells were gently washed with Phosphate Buffer Saline (PBS) for 3 times; then, 3.7% Paraformaldehyde solution was added for fixing, for 5 min, the cells were permeabilized with 1% Triton X for 10 min and colored with Texas Red-Phalloidin for 40 min (at dark). The cells were gently washed with PBS after each step. The visualization of the as-prepared samples was performed using an Olympus LX71 fluorescence microscope and the image recording was performedusing an ixon+ image recorder (Andor Technology).

Values were presented as means ± standard error of the mean. Data werestatistically analyzed using a two-tailed Student’s test, with *p* ≤ 0.05 accepted as statistically significant.

#### 3.4.2. *In Vitro* Antibacterial Activity

The antibacterial potential of the experimentally modified wound dressings was assessed *in vitro* against two clinically relevant bacterial strains that are often incriminated in nosocomial infections, namely the Gram-positive species *Staphylococcus aureus* ATCC 25923 and *Pseudomonas aeruginosa* ATCC27853. The strains are maintained as glycerol stocks in the culture collection of Microbiology Immunology Department of Faculty of Biology, University of Bucharest. The antibacterial efficiency was assessed by considering the bacterial development and colonization inthe presence of regular polyester-nylon dressings (positive control) and nanosilver-coated wound dressings. Thus, bare and AgNPs-based wound dressing sections were placed in 6-wellplates, followed by the inoculation of 2 mL of microbial suspension of 0.5 McFarland standard density (1.5 × 10^8^ CFU/mL) from each bacterial strain obtained directly into sterile broth medium. Subsequently, the inoculated plates were incubated for 24 h at 37 °C. Thereafter, the culture medium was removed and the specimens were washed with sterile phosphate buffered saline (PBS). The wound dressing sections (both uncoated and nano-modified) were afterwards placed in fresh medium and incubated at 37 °C for 24 and 48 h. After incubation, the wound dressing samples were gently washed with sterile phosphate buffered saline and further placed in 1.5 mL centrifuge tubes containing 750 μL of PBS. The as-obtained specimens were centrifuged for 30 s and subsequently subjected to ultrasounds for 10 s. Serial ten-fold dilutions were performed and distributed on Petri dishes containing Luria agar, for viable cell counts assay. All the experiments were performed in triplicate and repeated in three separate occasions.

#### 3.4.3. *In Vivo* Biocompatibility and Biodistribution of Nanostructures

The experimental protocol was applied according with the European Council Directive No. 86/609 (24 November 1986), the European Convention for the Protection of Vertebrate Animals used for Experimental and Other Scientific Purposes (2 December 2005), and the Romanian Parliament Law No. 43 (11 April 2014) on the protection of animals used for scientific purposes. The study was approved by the Ethics Committee of the University of Medicine and Pharmacy of Craiova, Romania (Approval Report No. 118/27.05.2015).

Three weeks old BALB/c mice were aseptically injected with 100-μL of 1 mg/mL dispersion of nanostructures, obtained in saline and previously sterilized by UV irradiation for 30 min. Intravenous administration was carried out slowly, under general anesthesia (Ketamine/Xylazine mixture), into the left jugular vein, using a catheter. Reference mice were injected with 100-μL of saline. The mice were kept in standard conditions before the organs removal. At 2 days and 10 days after the beginning of the experiment, the animals were euthanized, under general anesthesia, for the sampling of internal organs (brain, liver, myocardium, pancreas, lung, kidney and spleen).

Directly after the sampling, the biological material was washed in PBS to remove blood. Then, the internal organs were fixed in 10% buffered neutral formalin, for 72 h, at room temperature, and processed for routinely histological paraffin-embedding technique.

For the histological analysis of nanostructures, 4-μm thick serial sections were cut on a MICROM HM355s rotary microtome (MICROM International GmbH, Walldorf, Germany) equipped with a waterfall-based section transfer system (STS, MICROM). The cross-sections were placed on histological blades treated with poly-l-Lysine (Sigma-Aldrich, Munich, Germany). After Hematoxylin–Eosin (HE) classical staining, cross-sections were evaluated and photographed using a Nikon Eclipse 55*i* light microscope equipped with a Nikon DS–*Fi*1 CCD high definition video camera (Nikon Instruments, Apidrag, Romania). Images were captured, stored and analyzed using Image ProPlus 7 AMS software (Media Cybernetics Inc., Marlow, Buckinghamshire, UK) [36,37,38].

## 4. Conclusions

The selected chemical synthesis method represents a fast, simple, efficient and non-toxic *in situ* method to experimentally synthesize pure and crystalline silver nanoparticles, which can be utilized in biomedical and pharmaceutical purposes (*i.e.*, for the coating of commercial wound dressings). The structural and topography-related analysis showed a good distribution of the AgNPs onto the fibers of the polyester-nylon wound dressings, without any changes on the polymeric surface of the dressings. Furthermore, *in vitro* and *in vivo* tests showed a very good biocompatibility and biodistribution of these nanoparticles within a mouse model. Our data revealed that the nanoparticles do not persist within the body more than few days (after ten days, they could only be detected within the spleen) and do not enter vital organs such as brain, heart and pancreas. The *in vitro* assessment of antibacterial activity of the NanoAg-WDs revealed distinctive kinetics regarding the cellular proliferation and biofilm development of the selected bacterial strains, in the presence of regular and silver-containing wound dressings. Significant results were obtained against both Gram-negative and Gram-positive bacteria, but the most extensive clinical relevance correlates with an enhanced antibacterial efficiency against *P. aeruginosa*. Considering the previously reported and discussed results, we conclude that the main goal of this research was achieved by obtaining silver nanoparticles for improved biomedical surfaces and devices, with increased antibacterial efficiency, optimized for the commercial available bioactive dressings.
